# *Staphylococcus aureus* Sepsis Induces Early Renal Mitochondrial DNA Repair and Mitochondrial Biogenesis in Mice

**DOI:** 10.1371/journal.pone.0100912

**Published:** 2014-07-02

**Authors:** Raquel R. Bartz, Ping Fu, Hagir B. Suliman, Stephen D. Crowley, Nancy Chou MacGarvey, Karen Welty-Wolf, Claude A. Piantadosi

**Affiliations:** 1 Department of Anesthesiology, Duke University School of Medicine, Durham, North Carolina, United States of America; 2 Division of Nephrology, Department of Medicine, Duke University School of Medicine, Durham, North Carolina, United States of America; 3 Department of Medicine, Drexel College of Medicine, Philadelphia, Pennsylvania, United States of America; 4 Durham Veterans Affairs Medical Center, Durham, North Carolina, United States of America; 5 Division of Pulmonary, Allergy, and Critical Care Medicine, Department of Medicine, Duke University School of Medicine, Durham, North Carolina, United States of America; National Cancer Institute, United States of America

## Abstract

Acute kidney injury (AKI) contributes to the high morbidity and mortality of multi-system organ failure in sepsis. However, recovery of renal function after sepsis-induced AKI suggests active repair of energy-producing pathways. Here, we tested the hypothesis in mice that *Staphyloccocus aureus* sepsis damages mitochondrial DNA (mtDNA) in the kidney and activates mtDNA repair and mitochondrial biogenesis. Sepsis was induced in wild-type C57Bl/6J and *Cox-8* Gfp-tagged mitochondrial-reporter mice via intraperitoneal fibrin clots embedded with *S. aureus*. Kidneys from surviving mice were harvested at time zero (control), 24, or 48 hours after infection and evaluated for renal inflammation, oxidative stress markers, mtDNA content, and mitochondrial biogenesis markers, and OGG1 and UDG mitochondrial DNA repair enzymes. We examined the kidneys of the mitochondrial reporter mice for changes in staining density and distribution. *S. aureus* sepsis induced sharp amplification of renal *Tnf, Il-10*, and *Ngal* mRNAs with decreased renal mtDNA content and increased tubular and glomerular cell death and accumulation of protein carbonyls and 8-OHdG. Subsequently, mtDNA repair and mitochondrial biogenesis was evidenced by elevated OGG1 levels and significant increases in NRF-1, NRF-2, and mtTFA expression. Overall, renal mitochondrial mass, tracked by citrate synthase mRNA and protein, increased in parallel with changes in mitochondrial GFP-fluorescence especially in proximal tubules in the renal cortex and medulla. Sub-lethal *S. aureus* sepsis thus induces widespread renal mitochondrial damage that triggers the induction of the renal mtDNA repair protein, OGG1, and mitochondrial biogenesis as a conspicuous resolution mechanism after systemic bacterial infection.

## Introduction

Multi-system organ failure in severe sepsis results in significant morbidity and mortality in patients requiring intensive care. The kidneys are among the major damaged organs that are independently associated with death [1]–[3]. Acute kidney injury (AKI) and renal dysfunction occurs not only during sepsis, but accompanies many other confounding insults including ischemia-reperfusion and other causes of oxidative and nitrosative stress, as well as nephrotoxic agents [1], [2], [4]. Previously AKI in sepsis was thought to mainly reflect decreased renal blood flow and ischemia-reperfusion; however, recent challenges to this paradigm have offered more complex and multifactorial inciting events [5]–[8]. Pathologically, nephrotoxic injuries often appear similar, but in each case, little is known about the molecular responses leading to renal recovery and how the mitochondrial quality control process is involved in renal repair mechanisms.

The kidney is highly metabolic and requires considerable energy in the form of ATP to functionally regulate fluid and electrolyte balance. During oxidative phosphorylation, in addition to ATP, the mitochondrial electron transport chain produces reactive oxygen and reactive nitrogen species (ROS/RNS) [9]–[11]. Concomitantly, the innate immune response and early-phase cytokine production, independently of ischemia-reperfusion injury, also increases mitochondrial ROS/RNS production, which contributes to cell damage, organ failure and immune dysregulation after exposure to infectious and toxic agents [12]. For instance, renal ROS/RNS production during endotoxemia stimulates TLR4 receptors in S1 kidney proximal tubules and induces TNF-a secretion resulting in oxidative stress in downstream S2 and S3 tubules [13], [14]. Furthermore, excess TNF-a also leads to apoptosis of bovine glomerular endothelial cells [15]. In experimental sepsis, cytokine-dependent ROS and RNS production leads to oxidized and nitrosated proteins, lipids, and nucleic acid products in many organs [16], [17].

During acute inflammatory states, cells up-regulate anti-oxidant responses in order to limit intracellular damage from ROS/RNS, maintain cellular viability and integrity, and prevent apoptosis. Moreover, excessive intracellular ROS/RNS production without a compensatory, balanced anti-oxidant response, for example in the elderly, promotes further inflammation and leads to macromolecular damage including mitochondrial DNA (mtDNA) damage, lipid peroxidation, and protein carbonyl production [17], [18]. Among the mechanisms that mitigate ROS/RNS damage are the up-regulation of targeted antioxidant enzymes such as catalase, peroxidases, and superoxide dismutases (SOD). Induction of mitochondrial SOD2 or other protective enzymes by various pharmacological agents can effectively prevent kidney injury *in vitro* and *in vivo* [19], [20].

A correlation between intracellular ROS/RNS and high levels of mtDNA damage is found when intracellular antioxidant proteins do not respond adequately or enzymes that remove the mtDNA-damaged nucleotides are ineffective or overwhelmed. A widely studied oxidative mtDNA product, 8-OH-deoxyguanosine or 8-OHdG, is incorporated into mtDNA and can lead to GC:TA transversions as well as to ineffective transcription [21], [22]. However, 8-OHdG products can be effectively repaired by the enzyme 8-hydroxyguanine DNA glycosylase (OGG1), a glycase that is functionally important in mtDNA repair [23]. We previously found an important role for OGG1 in mtDNA 8-OHdG removal in the liver during sepsis and associated its gene transcription with the regulation of nuclear genes required for mitochondrial biogenesis [16]. Given that renal dysfunction is prominent in *Ogg1*-deficient mice after exposure to potassium bromate, a moderate oxidizing agent [24], we hypothesize the enzymes importance during AKI from a septic insult.

Therefore, we sought to determine if the mtDNA repair enzyme system was up-regulated in the kidneys in response to *S. aureus,* a major cause of severe sepsis and whether the kidney’s program for mtDNA repair and mitochondrial biogenesis accompanies the host immune response. Previous investigators have shown that oxidants induce peroxisome proliferator-activated receptor co-activator 1-a (PGC-1a) expression in conjunction with return of intracellular respiration and mitochondrial biogenesis in highly metabolic renal proximal tubule cells and that mice with a proximal tubule specific *Ppargc1a* deletion compared to litter-mate controls demonstrated persistent renal cell injury in response to endotoxin challenge [25], [26]. This co-activator may also have an important in role in the regulation of mtDNA repair however, this has not yet been tested. Here, we report that *S. aureus* sepsis causes significant early oxidative damage to mtDNA in the mouse kidney and that mtDNA repair enzymes are promptly induced and recruited to renal mitochondria during sepsis. Moreover, we demonstrate a relatively early sepsis-induced increase in the genetic program of mitochondrial biogenesis in the kidney which localizes strongly to the proximal tubules.

## Methods and Materials

### Animals

All animal protocols were approved by the Duke IACUC. *S. aureus* sepsis was induced in male C57Bl/6J (12–18 weeks old) and male transgenic GFP-COX8 (12 weeks old) mitochondrial reporter mice by intraperitoneal instillation of *S. aureus* 10^7^ CFU in a 1.5 ml fibrin-clot as described [27]. Mice were given 1.0 ml of 0.9% NaCl and monitored every 6 hours for up to 48 hours after clot implantation. Control mice were euthanized with isoflurane and aortic transection at time zero and the kidneys removed for comparison with the post-sepsis mice. Healthy mice were anesthetized with ketamine (50 mg/ml), xylazine (20 mg/ml), and given Buprenex 0.8 mg IP for pain control followed by sterile midline laparotomy and fibrin-clot was placement. The incision was closed with proline sutures. Because this was a survival study mice were sacrificed at 24 and 48 hours after fibrin-clot deposition with isoflurane followed by aortic transection. This *S. aureus* inoculation dose, similar to severe human sepsis, results in approximately 30–50% survival by seven days based on prior studies. Kidneys were either removed, snap-frozen, and stored in –80°C until biochemical analysis or divided and immersed in 10% formalin for 24 hours at room temperature followed by 70% ethanol (EtOH) at 4°C. The tissue blocks were embedded in paraffin and sectioned into 4 micron slices on glass slides. These slides were utilized for immunofluorescence, TUNEL staining, or hematoxylin and eosinophilic (H&E) staining.

### Renal Histology and immunohistochemistry

For immunohistochemistry, formalin-fixed, paraffin-embedded renal sections were immuno-labeled with primary antibodies to 8-OHdG or OGG1 followed by fluorescent-labeled secondary antibodies. For H&E staining paraffin-embedded sections on slides were immersed in water and then dipped into Mayer’s hematoxylin for 30 seconds. These slides were rinsed in water and stained with 1% eosin Y solution for 30 seconds. They were dehydrated with 95% alcohol and then 100% alcohol. The alcohol was extracted with xylene.

### Analysis of blood samples

At time zero, 24 hours, and 48 hours after infection, blood was drawn from the IVC into 200 microliter vials and the plasma was isolated and analyzed for sodium, potassium, chloride, bicarbonate, blood urea nitrogen, and creatinine (Abaxis, VetScan).

### Mitochondrial DNA analysis

Cellular DNA was extracted using Genelute mammalian genomic DNA extraction kit (Sigma, St. Louis, MO). Mitochondrial DNA was quantified by SYBR green quantitative PCR of cytochrome c oxidase subunit 1 and normalized to nuclear-encoded 18S.

### Protein Immunoassays:

Kidney tissue was sub-fractionated into either nuclear or mitochondrial compartments using previously described techniques [28]. Whole cell, crude mitochondrial, or nuclear protein lysate fractions were used for Western blot analysis. To quantitate mitochondrial-specific proteins, mitochondrial fractions were obtained from renal homogenates [29]. For Western blot analysis, 20 µg of assayed protein samples were resolved by sodium dodecyl sulfate-polyacrylamide gel electrophoresis (SDS-PAGE) using 8% or gradient gels (Biorad, Hercules, CA). The separated protein was transferred to Immobilin P membranes (Millipore, Billerica, MA) and blocked with 4% nonfat dry milk in TBST (Sigma). Membranes were incubated overnight at 4°C with antibodies to OGG1 (1∶1000), uracil DNA glycosylase (UDG) (1∶1000), superoxide dismutase 2 (SOD2) (1∶1000), transcription factor A, mitochondrial (mtTFA) (1∶1000), nuclear respiratory factor-1 (NRF-1) (1∶1000), nuclear respiratory factor–2 (NRF-2) (1∶1000), nuclear factor erythroid 2-related factor 2 (NFE2l2) (1∶1000), PGC-1α (1∶1000), thioredoxin 2 (TRX2) (1∶1000), polymerase g (POLg) (1∶1000), heme oxygenase 1 (HO-1) or citrate synthase (CS) (1∶1000). After application of primary antibodies, each membrane was washed with TBST and incubated with horseradish peroxidase-conjugated anti-rabbit or anti-goat antibody, washed again, and developed using ECL chemiluminescence reagents (Amersham, GE Healthcare, Piscataway, NJ). The blots were digitized and the protein density quantified in the mid-dynamic range relative to tubulin or porin (BioRad ImageQuant Software, Hercules, CA).

### Real-time RT-PCR:

qRT-PCR was performed using an ABI prism 7000 Sequence Detection System with TaqMan gene expression and premix assays for tumor necrosis factor (Tnf), Mm00443258_m1; Tfam, Mm0114667_m1; Ogg1, Mm01184571_g1; Nrf-1, Mm01135607_m1; NRF-2 (Gabpa) Mm00484597_m1, Neutrophil gelatinase-associated lipocalin (Ngal) Mm01324469_g1, Interleukin-10 (Il-10) Mm 00439616_m1, Peroxisome proliferator-activated receptor coactivator 1-a (Ppargc1a) Mm 00447187_g1 from Applied Biosystems, Foster City, Ca. 18S rRNA was used as an endogenous control. Quantification of gene expression was determined by the comparative threshold cycle CT and relative quantification (RQ) method.

### TdT-mediated dUTP-biotin nick end-labeling (TUNEL) assay

TUNEL assay was performed on paraffin-embedded kidney sections after incubating the slides for 10 min at 70°C in assay buffer. After sequential rehydration with xylene, graded EtOH and water, the sections were treated with 10 mM Tris-Cl and incubated with proteinase K at room temperature. Slides were washed and incubated with 3% methanol for 30 minutes, rinsed in ultrapure water and incubated in elongation buffer for 60 minutes at 37°C. The slides were rinsed three times in ultrapure water and then in termination buffer (300 mM NaCl/30 mM sodium citrate) for 15 minutes at room temperature. Sections were sequentially rinsed in PBS, 2% BSA and PBS at room temperature, then incubated with ExtrAvidin-peroxidase for 15 minutes then rinsed in 2% BSA and PBS at least four times. Sections were stained with AEC for 30 minutes at 37°C and rinsed in ultrapure water before drying, mounting and photography.

### Statistical analysis

Grouped data are expressed as mean + standard deviation. Each time point analysis contained at least three samples from three mice per group. Differences between groups were determined by one-way analysis of variance followed by Tukey post-test analysis using Sigma Stat and Sigma Plot software. (Systat Software Inc., Chicago, IL). *P*<0.05 was considered statistically significant.

## Results

### Systemic *Staph. aureus* sepsis induces renal inflammation and cell damage in mice

**Figure 1:**
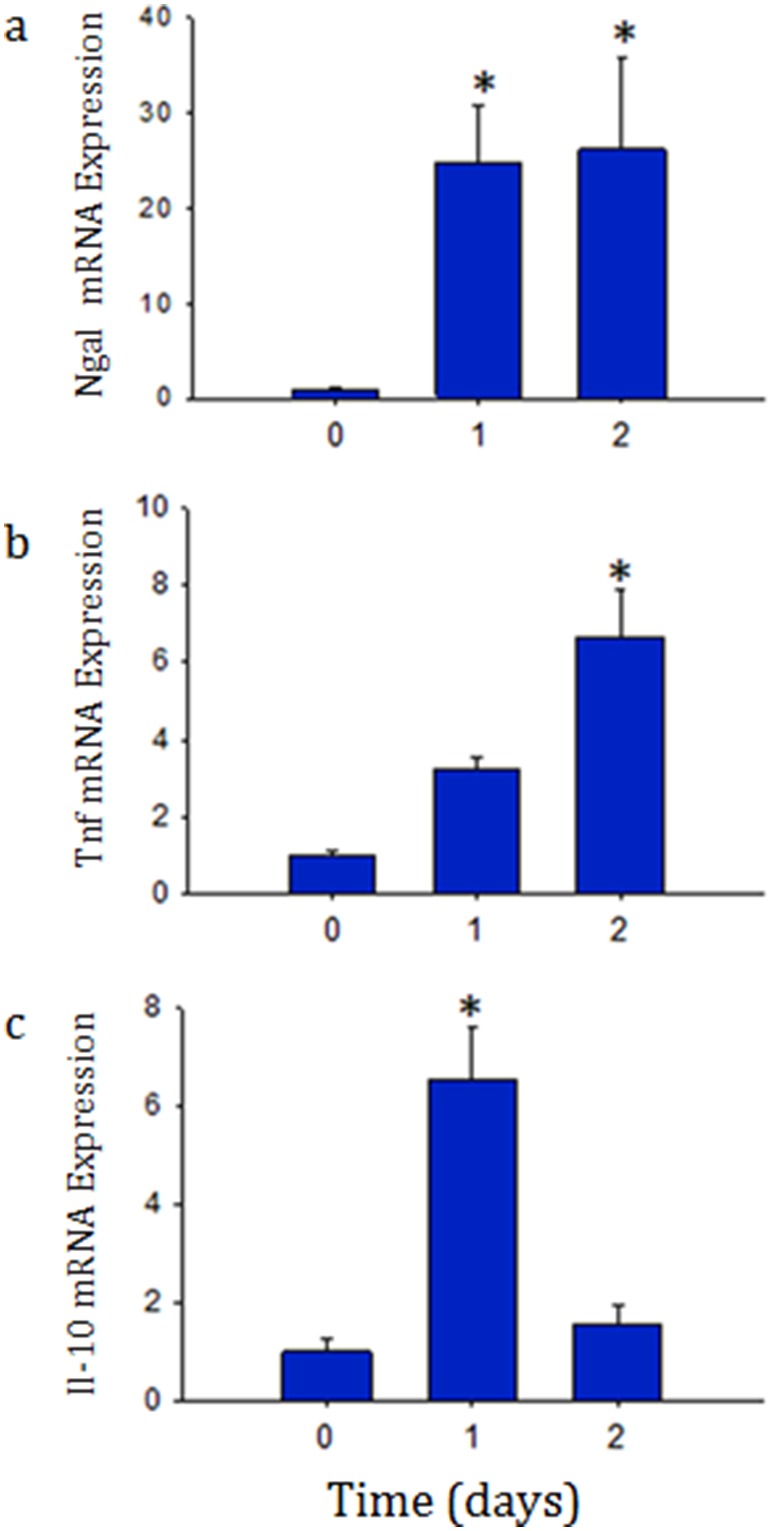
a, b, and c: Profiling of renal tissue molecular inflammatory markers using Real-time PCR after *S. aureus* sepsis. Quantitative real-time PCR for Ngal (a), Tnf (b), and Il-10 (c) mRNA in renal tissue at time zero (control), Day 1 (1), and Day 2 (2) after infection. Ngal mRNA increased more than 20-fold over control by Day 1 of infection, Tnf, and Il-10 also increased significantly, however at different time points. Data represents 6 mice in each group. *P<0.05 is considered significant by ANOVA and Tukey’s post-test analysis.

**Figure 2:**
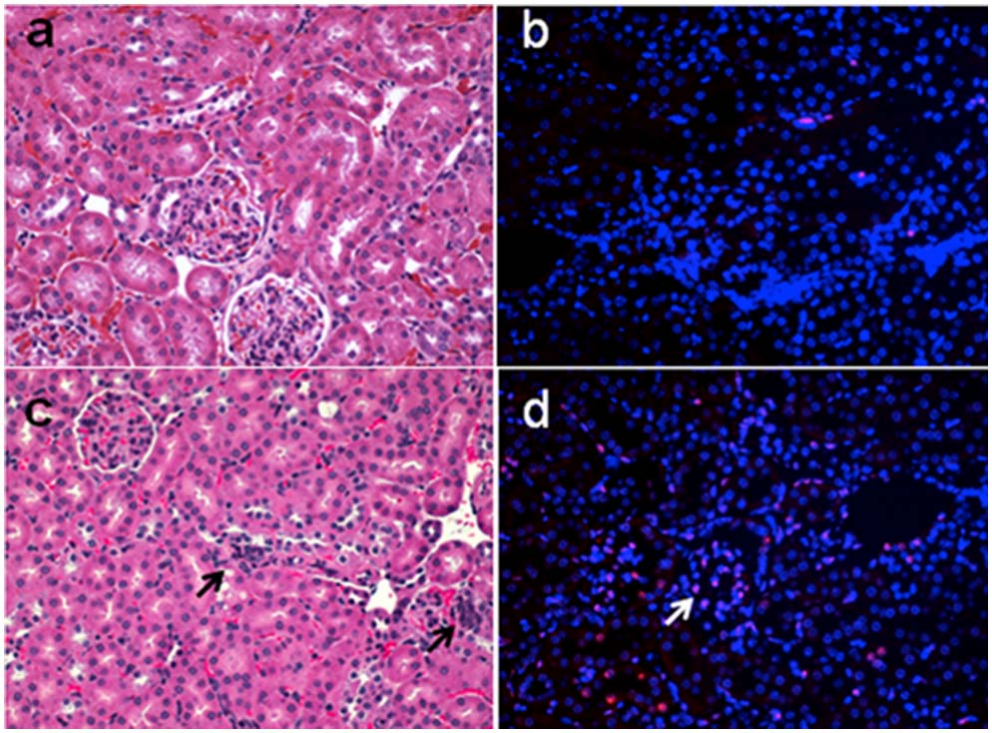
a, b, c, d: Representative photomicrographs of H&E staining of the renal cortex at time zero (a) and Day 1 (b) and TUNEL staining at time zero (c) and Day 1 after infection (d) after infection. Arrows indicate areas of necrosis/apoptosis in the H&E stain and TUNEL labeled nuclei. Note inflammatory cell infiltration.

**Figure 3:**
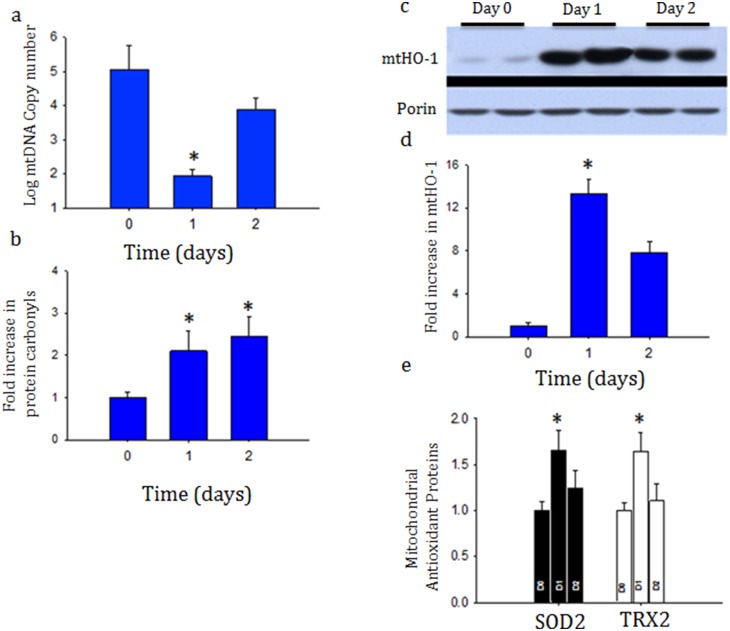
a, b, c, d and e: Mitochondrial DNA copy number based on real-time PCR of cytochrome B/18S levels from kidney (3a) after *S. aureus* infection. MtDNA decreased only transiently suggesting active repair and biogenesis. Oxy-blot analysis of whole-cell protein carbonyl content of whole kidney after *S. aureus* sepsis (3b). Western blot analysis and densitometry of heme-oxygenase 1 (HO-1) levels in the mitochondrial fraction (3c and 3d). Mitochondrial antioxidant enzymes levels of superoxide dismutase (SOD2) and thioredoxin (TRX2) relative to porin densitometry analysis (3e). Data represents 6 mice in each group. *P<0.05 is considered significant by ANOVA and Tukey’s post-test analysis.

The peritoneal inoculation of mice with *Staph. aureus* caused a rapid increase in renal inflammation shown by increased Ngal, Tnf, and Il-10, mRNA expression (Fig. 1a, 1b, and 1c). Ngal was chosen given it has been previously found to increase earlier than creatinine. Concordantly, examination of the kidneys by hematoxylin and eosin (H&E) and TUNEL staining revealed many focal areas of cortical necrosis and cell death (Fig. 2a, 2b, 2c, and 2d). However, there were no significant increases in BUN or creatinine over the 48 hour period (data not shown). These findings are consistent with well preserved clearance function. MtDNA, which is poorly protected and susceptible to oxidative damage was severely affected in the kidneys, shown by a transient decrease in mtDNA content, which was restored towards baseline by 48 hours suggesting active repair mechanisms (Fig. 3a).

### Oxidative stress damages mitochondrial DNA, but is balanced by corresponding cytoprotective mechanisms

**Figure 4:**
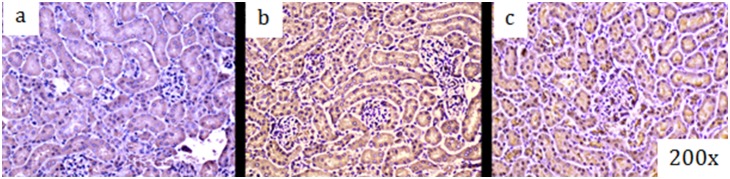
a, b, and c: Immunohistochemistry, brown staining, for 8-0H8dG in kidney before (4a), 24 hrs (4b) and 48 hrs (4c) after *S. aureus* peritoneal sepsis in mice.

Mitochondrial protein carbonyls, a marker of oxidative stress, also increased in the renal tissue (Fig. 3b). In addition, the cytoprotective enzyme, HO-1, accumulated impressively in the mitochondrial fraction of homogenized renal tissue (Fig. 3c and d). A response to mitochondrial oxidative stress was also revealed by an increase in renal SOD2 and TRX2 protein levels (Fig. 3e). The 8-OHdG adduct, a well-studied product of mtDNA oxidation is particularly biologically relevant because, unless it is repaired, may lead to mutations or dysfunctional mtDNA genome. We evaluated the oxidized DNA product in the kidney during sepsis by immunohistochemistry and found that 8-OHdG increased from control to 48 hours as shown by increased brown staining after infection specifically in cells localized to punctate structures in cytoplasmic compartment (Fig. 4a, 4b, 4c) suggesting mitochondrial localization.

### Mitochondrial DNA repair mechanisms are up-regulated

**Figure 5:**
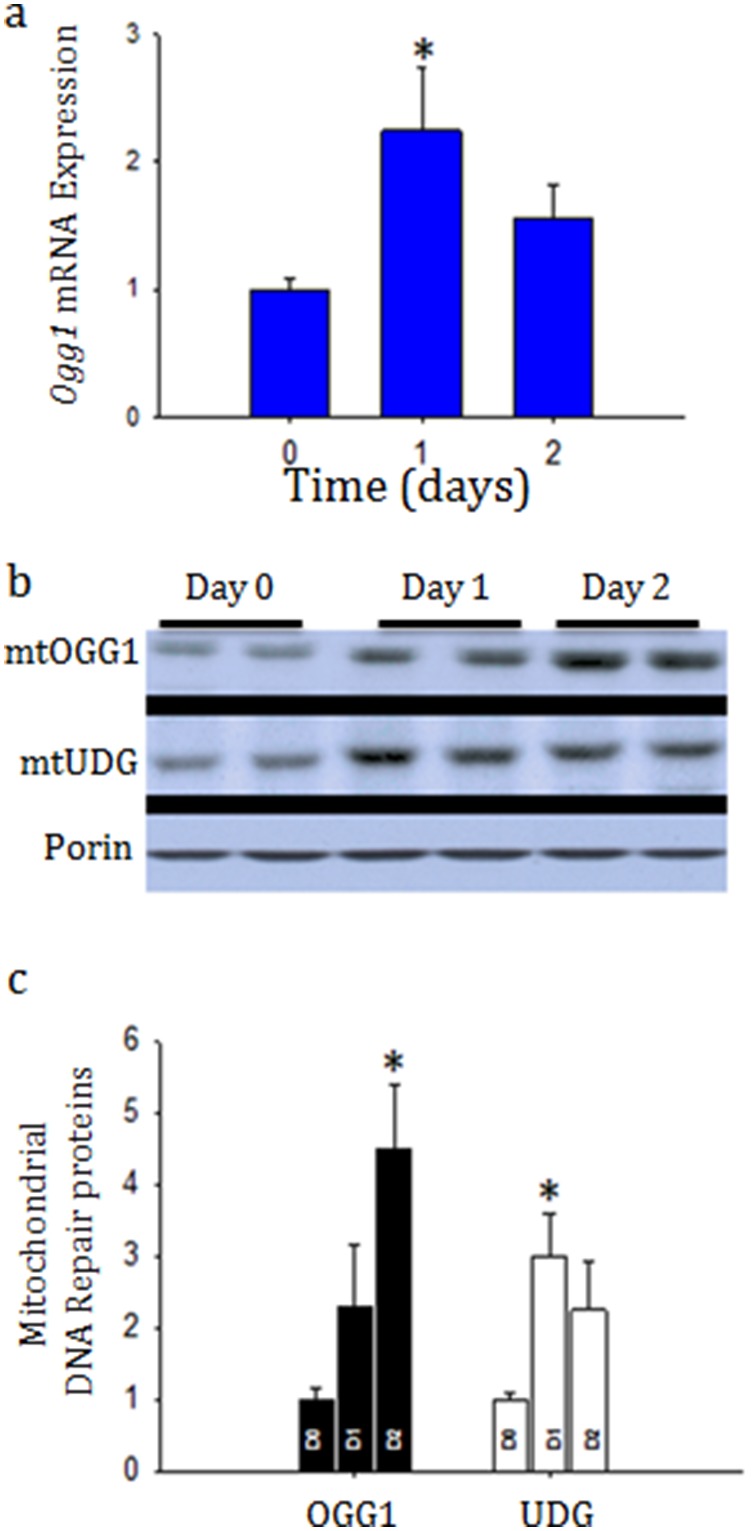
a, b, and c: Determination of mitochondrial DNA repair enzymes after sepsis in the crude mitochondrial fraction. Figure 5a shows quantitative Real-time PCR for Ogg1, a base-excision repair protein especially important for repair of mitochondrial DNA, from whole kidney. Representative western blot analysis for OGG1 and UDG, mitochondrial DNA repair enzymes, compared to Porin from the mitochondrial fraction at control, and at Day 1 and Day 2 after sepsis (5b) and relative densitometry compared from three mice at each time-point (5c). OGG1 increases significantly in the mitochondrial fraction. *P<0.05 significant by ANOVA and Tukey’s post-hoc analysis.

**Figure 6:**
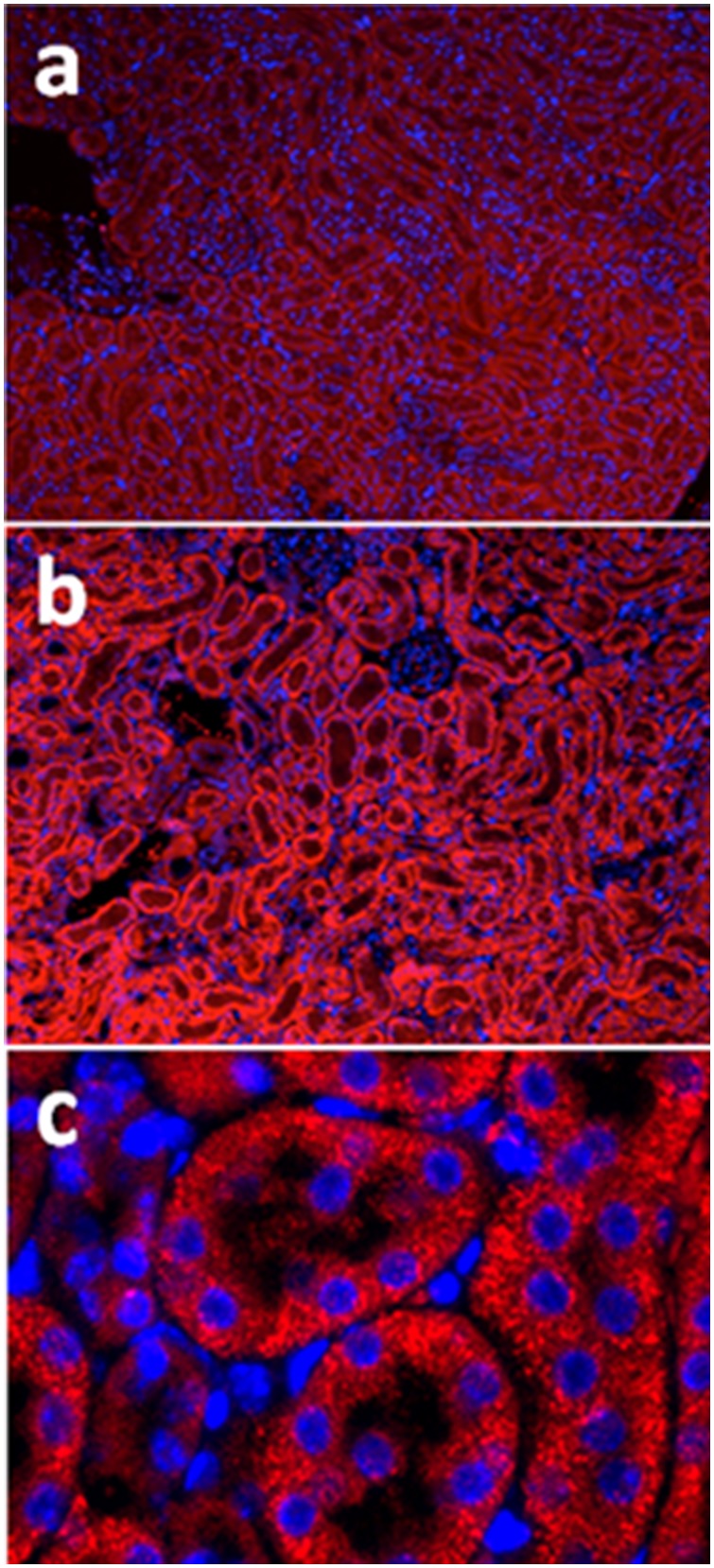
a, b, and c: Representative immunofluorescence for OGG1 (red) in renal tissue before and after infection at time 0 (6a) and Day 2 (6b) after infection at 10x and 40x magnification (6c). OGG1 increased in the kidneys by immunofluorescence confirming mRNA and Western results.

Due to the significant early renal oxidative damage observed during sepsis, we examined changes in the mtDNA base-excision repair enzymes, Ogg1 mRNA and OGG1 protein expression in the kidney as well as UDG, another mitochondrial DNA repair enzyme, protein expression. Indeed, renal Ogg1 mRNA expression were both significantly increased by 24 h after infection and associated with accumulation of both enzymes in the mitochondria (Fig. 5 a–c and 6 a–c).

### *S. aureus* sepsis induces mitochondrial biogenesis in the kidneys

**Figure 7:**
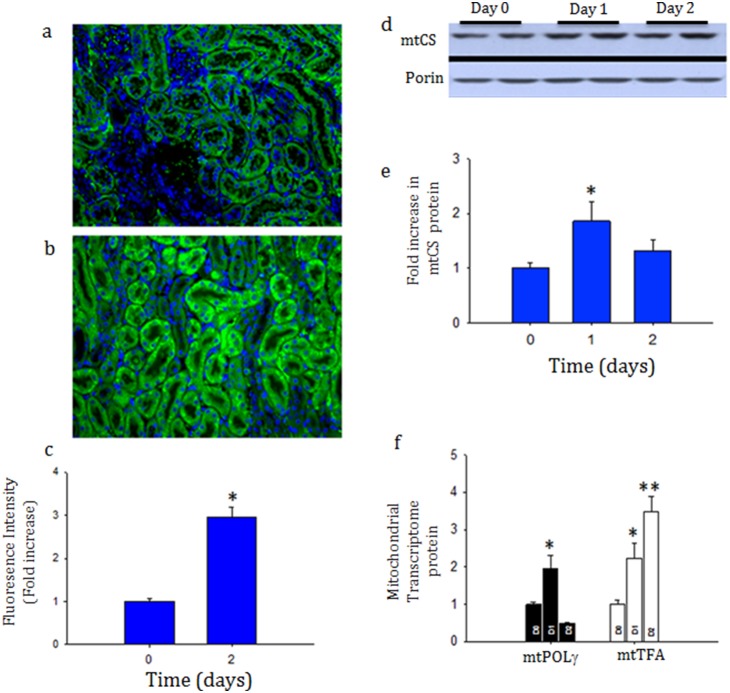
a, b, c, d, and e: Evidence of mitochondrial biogenesis after sepsis in GFP-tagged mitochondrial *Cox8* subunit mice. Representative immunfluorescence of renal cortex at time zero (7a) and 48 hours (7b) after infection with *S. aureus* Figure 7c shows the fluorescence intensity of each cortical area. The mitochondrial transcriptome increased after sepsis induction within the mitochondrial compartment as measured mitochondrial citrate synthase (mCS) (7d and 7e) by POLg and mtTFA (7f). Each time-point represents 3 mice. *P<0.05 significant by ANOVA and Tukey’s post-test analysis.

To determine if this DNA damage response is coordinated with mitochondrial biogenesis as in the liver [30], mitochondrial biogenesis was tracked in *Cox8*-GFP transgenic mitochondrial reporter mice. *S. aureus.* infection increased mitochondrial mass within the kidney characterized by elevated citrate synthase and mitochondrial GFP fluorescence (Fig. 7a–e). The increase in mitochondrial mass was associated with significant increase in renal mtTFA and POLg protein, both of which are required for mtDNA transcription and replication (Fig. 7f).

**Figure 8:**
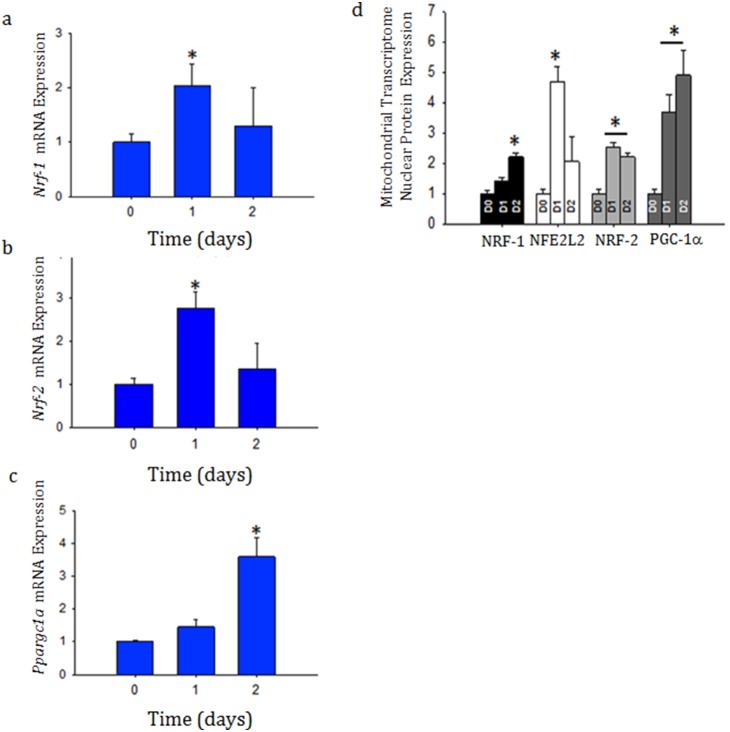
a, b, c, and d: Detection of nuclear markers of mitochondrial biogenesis. Whole kidney mRNA was evaluated by quantitative real-time PCR for Nrf-1 (8a), Nrf-2 (Gabpa) (8b), and Ppargc1a (8c) at time zero, day 1 and day 2 after infection as well as kidney nuclear protein densitometry for NRF-1, NFE2l2, NRF-2 (GABPa), and PGC-1a relative to histone (8d) by western blot analysis. Each time-point represents 3 mice. *P<0.05 significant by ANOVA and Tukey’s post-test analysis.

Renal mitochondrial biogenesis was further evaluated by assessing NRF-1, NRF-2 (GABPa), and PGC-1a at both the mRNA and protein levels (Fig. 8 a–d). Given that a large amount of ATP is required for kidney cortical function, Nrf-1 mRNA and NRF-1 protein was constitutively expressed before sepsis, but it also increased significantly during sepsis (Fig. 8a and 8d). We also found that Nrf-2 (Gabpα) mRNA and NRF-2 protein increased at 48 hours after infection (Fig. 8b and 8c) and the protein increased significantly in the nucleus (Fig. 8d). NRF-1 and NRF-2 are both PGC-1a partners, and Ppargc1a mRNA and protein increased significantly as well (Fig. 8c and d). Because we found renal oxidative stress to be prominent in response to sepsis we also probed for NFE2L2, a transcription factor important in the anti-oxidant response, and found that it too was increased in the nuclear compartment during sepsis at 24 hours (Fig. 8d).

## Discussion

Acute kidney injury is a major complication of severe sepsis that increases ICU length of stay, the need for dialysis, and mortality, especially in elderly patients [31]. Because the cellular mechanisms of sepsis-induced renal dysfunction are not completely understood [32], we sought experimentally to address the hypothesis that sepsis–induced oxidative stress in the kidney results in early mtDNA damage, which then leads to active up-regulation of mtDNA repair enzymes and subsequent mitochondrial biogenesis. The capacity to induce mtDNA repair and mitochondrial biogenesis are vital for organ homeostasis, for survival, and for recovery from cell damage [33], [34]. We found that *S. aureus* sepsis leads to *in vivo* renal mtDNA damage and to recruitment of mtDNA repair enzymes into renal mitochondria. We also found that the renal mitochondrial biogenesis transcriptional program is rapidly inducible in sepsis. This work introduces several potential novel targets to help prevent sepsis-induced AKI, mortality from MODS and to improve the rate of resolution of the renal injury.

The structural and molecular profiling of renal tissue in this study, strongly suggest that mitochondrial dysfunction is a key contributor to kidney impairment that occurs during sepsis. Previously, in the liver, we found that transcription factors, NRF-1 and NRF-2, bind to consensus promoter sequences in *Ogg1* suggesting this is part of larger program of mtDNA repair and mitochondrial biogenesis in response to infection [16]. Here we found that early mtDNA damage produces a robust repair response and induces early mitochondrial biogenesis in the kidneys despite a lack of early elevations in BUN and creatinine. However, the increases in PGC-1a and NRF-1 in the kidney were delayed in sepsis compared to the liver and heart. Due to the complexity of PGC-1a regulation and the range of molecular events that occurs during sepsis, the identification of a basic mechanism for the later expression of PGC-1a and NRF-1 will be challenging, but our current data do suggest that mtDNA repair and mitochondrial biogenesis are induced before the detection of loss of renal function by usual BUN/Cr measurements and likely represent kidney cell pro-survival mechanisms during severe sepsis. This actual relationship between mtDNA repair and loss of GFR will require specific studies of renal filtration.

The kidney is highly dependent on mitochondrial ATP generation to move solutes against electrochemical gradients [35], [36]. Excessive mtDNA oxidation must eventually compromise these processes, as well as lead to inhibition of the replication of the mitochondrial genome that is required for effective mitochondrial biogenesis. Others have recently shown by postmortem examination that liver and kidney mitochondrial damage occurs in patients who die from sepsis and that these cells undergo autophagy or mitophagy [37], [38]. Accordingly, mitochondrial biogenesis and mitophagy is required for cellular repair during sepsis in both mice and humans and the inability to properly activate these processes is associated with mortality [27], [38], [39].

The ability to repair mtDNA has also been shown to protect the kidney from oxidative stress. For instance, exposure to a strong oxidizing agent, potassium bromate (KBRO_3_), produces dramatic kidney damage in mice deficient in *Ogg1* compared with WT controls. The exposed *Ogg1* knockout mouse has more than tenfold greater oxidized DNA content as well as an increased numbers of apoptotic kidney cells by TUNEL assay compared to wild-type controls [24]. Furthermore, 8-OHdG accumulates in ischemia/reperfusion injury of rat kidneys and Ogg1 mRNA is found to increase in the renal cortex over a 7 day period [40]. Prior investigations in rat lung have found that targeting OGG1 to the mitochondria leads to decreased mtDNA oxidative damage and improved endothelial integrity [41] as well as to decreased apoptosis in pulmonary artery endothelial cells in response to oxidant injury [42]. Taken together, these data suggest that mtDNA repair is crucial to maintain renal cellular function and protection during the oxidative stress of sepsis. However, the degree to which renal cells remain functional during periods of active mtDNA repair in response to sepsis is an open question, as they may revert to a state of metabolic stasis during repair.

Among the other cytoprotective enzymes, we found that the renal mitochondrial protein fraction contained detectable HO-1 and significantly increased levels of SOD2. This is consistent with the finding in mice that are exposed to endotoxin, a TLR-4 ligand, which also leads to an increase HO-1 expression in the S1 tubules as well is in response to cisplatin-induced and HgCl_2_ nephrotoxicity [43], [44]. It has also been shown that mitochondrial targeted HO-1 decreases oxidant stress in renal epithelial cell and prevents excessive heme accumulation and ROS generation *in vitro* [45].

Toxin-induced oxidative stress has been shown to cause mitochondrial dysfunction in renal proximal tubular cells (RPTC) and the program of mitochondrial biogenesis is up-regulated, eventually leading to intracellular repair [25], [46]. Furthermore, pharmacological induction of mitochondrial biogenesis by SRT1720 in RPTC results in activation of PGC-1aand increased mtDNA content under conditions of oxidative stress [47]. Also, Tran and colleagues have suggested that up-regulation of PGC-1a or induction of its activity may provide a mechanism for prevention and/or repair of acute kidney injury in mice subjected to LPS [26]. Although PGC-1a induction has not been previously shown to be linked to mtDNA repair, it is possible that PGC-1ainduction may lead to increase in OGG1, resulting in increased mtDNA repair, and renal protection against oxidative stress in sepsis. To prove this, however, requires future study.

In summary, systemic *S. aureus* sepsis damages renal mDNA in the mouse kidney, leading to induction of the nuclear program of mitochondrial biogenesis, which is required to maintain intracellular energy homeostasis and organ function during such periods of cell stress. Prior studies have suggested that mitochondrial biogenesis in the kidneys may improve the recovery of mitochondrial function during oxidative AKI, and accordingly, we find that renal inflammation and oxidative damage from *S. aureus* sepsis in mice triggers the mtDNA repair response and mitochondrial biogenesis *in vivo*, particularly in proximal renal tubule cells. The evidence presented here attaches importance to a better understanding of mtDNA repair mechanisms and mitochondrial biogenesis during severe sepsis in order to develop new therapeutic targets for renal protection in patients with sepsis.
